# The genome sequence of the Burnished Brass, *Diachrysia chrysitis* (Linnaeus, 1758)

**DOI:** 10.12688/wellcomeopenres.18990.1

**Published:** 2023-02-15

**Authors:** Douglas Boyes, University of Oxford and Wytham Woods Genome Acquisition Lab, Darwin Tree of Life Barcoding collective, Wellcome Sanger Institute Tree of Life programme, Wellcome Sanger Institute Scientific Operations: DNA Pipelines collective, Tree of Life Core Informatics collective, Peter W.H. Holland, Darwin Tree of Life Consortium

**Affiliations:** 1 UK Centre for Ecology and Hydrology, Wallingford, Oxfordshire, UK; 2 University of Oxford, Oxford, Oxfordshire, UK

**Keywords:** Diachrysia chrysitis, the Burnished Brass, genome sequence, chromosomal, Lepidoptera

## Abstract

We present a genome assembly from an individual male *Diachrysia chrysitis* (the Burnished Brass; Arthropoda; Insecta; Lepidoptera; Noctuidae). The genome sequence is 386 megabases in span. Most of the assembly is scaffolded into 31 chromosomal pseudomolecules, including the assembled Z sex chromosome. The mitochondrial genome has also been assembled and is 15.3 kilobases in length. Gene annotation of this assembly on Ensembl identified 18,320 protein coding genes.

## Species taxonomy

Eukaryota; Metazoa; Ecdysozoa; Arthropoda; Hexapoda; Insecta; Pterygota; Neoptera; Endopterygota; Lepidoptera; Glossata; Ditrysia; Noctuoidea; Noctuidae; Plusiinae; *Diachrysia*; *Diachrysia chrysitis* (Linnaeus, 1758) (NCBI:txid179674).

## Background

The Burnished Brass *Diachrysia chrisitis* (Linnaeus, 1758) is one of the most striking noctuid moths found in UK, characterised by shimmering metallic golden patches on the forewings. The adult is on the wing from June to September in the UK in two overlapping generations; the second generation has become more frequent since 1970 ( Randle *et al.*, 2019). Larvae feed primarily on nettle ( *Urtica dioica*) and sometimes other herbaceous plants. The species is widespread across the UK and found throughout much of Europe and Russia ( GBIF Secretariat, 2021).

The iridescent gold sheen on the forewings is a structural colour generated by light scattering and interference rather than a chemical pigment. Indeed, wing scales in the gold regions are devoid of melanin pigment and form a nanoscale multilayer structure enclosing a sandwich of irregular spheres ( Savić-Šević *et al.*, 2018). The spectrum of reflected light from golden regions of the wing is a close match to that generated by true metallic gold ( Pantelić *et al.*, 2017). These remarkable optical properties have stimulated efforts to mimic the structure in a laboratory setting, and a successful proof of concept has been achieved using layers of polysaccharide sandwiching spherical nanoparticles of variable size ( Savić-Šević *et al.*, 2018). Similar multilayer structures may ultimately prove useful in solar energy collection and other applications.

The typical form of the Burnished Brass moth has two separate bands of gold on the forewing separated by a broad region of brown scales, while a variant has a ‘bridge’ between the golden regions forming a letter ‘H’ pattern (form *juncta*). Although intermediate forms exist, there has been discussion over whether the two extreme wing pattern morphs in the UK represent different species, with the *juncta* form potentially being *D. stenochrysis*, a moth found across the Eastern Palaeartic Region and much of mainland Europe ( Hammond, 2022; Plant, 2010). Application of reflectance spectroscopy to wings of *D. chrisitis* and *D. stenochrysis* collected in Poland revealed significant differences related to chemical composition and scale structure, suggestive of species-level distinction ( Dyba *et al.*, 2022). These methods have not yet been applied to UK specimens. Molecular phylogenetic analysis using the mitochondrial COI gene also divides specimens from mainland Europe into distinct clades for *D. chrisitis* and *D. stenochrysis*. The taxonomic situation is less clear in the UK, since DNA barcodes from several UK specimens with the *juncta* wing pattern cluster with *D. chrisitis* rather than with *D. stenochrysis (*P.W.H. Holland and P.O. Mulhair analysis at the Barcode of Life Database ( BOLD, 2023)). This suggests either that there is a single species of burnished brass moth in the UK ( *D. chrysitis*) or that the two species exist but the *juncta* trait has introgressed across the species boundary. A recent preliminary report of UK moths with barcodes comparable to *D. stenochrysis* ( Hammond, 2022) needs to be followed up with further phylogenetic analyses and inclusion of more sequences from additional UK individuals, ideally from multiple genetic loci.

A genome sequence from *Diachrysia chrisitis* will prove useful as a reference genome for resolving the taxonomy of this genus and for probing the basis of species differentiation. It may also lay a foundation for understanding the developmental genetic basis of the unusual photonic scale structures. The complete genome sequence presented here is generated from an individual burnished brass moth with a *juncta* wing pattern and a *C. chrisitis*-type CO1 DNA barcode.

### Genome sequence report

**Figure 1.**
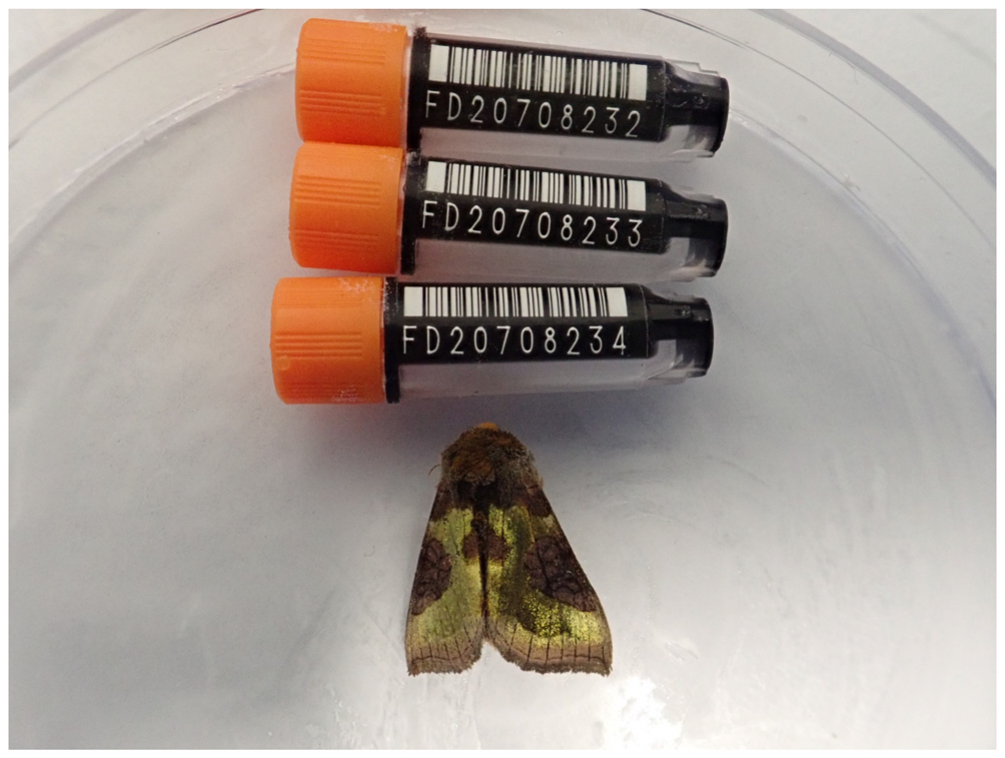
Photograph of the *Diachrysia chrysitis* (ilDiaChry1) specimen used for genome sequencing.

The genome was sequenced from one male *D. chrysitis* ( Figure 1) collected from Wytham Woods, UK (latitude 51.77, longitude –1.34). A total of 34-fold coverage in Pacific Biosciences single-molecule HiFi long reads was generated. Primary assembly contigs were scaffolded with chromosome conformation Hi-C data.

**Figure 2.**
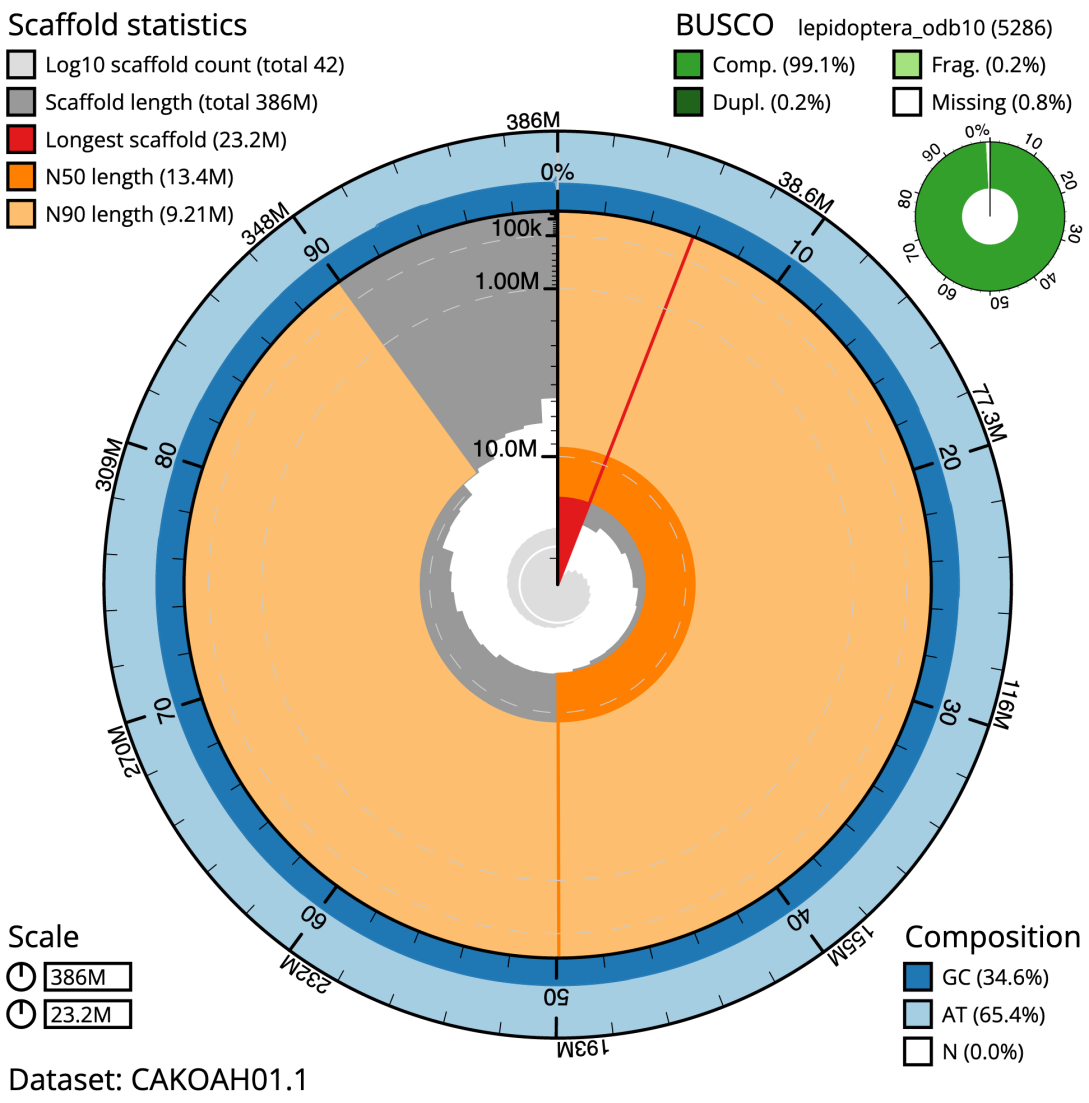
Genome assembly of *Diachrysia chrysitis*, ilDiaChry1.1: metrics. The BlobToolKit Snailplot shows N50 metrics and BUSCO gene completeness. The main plot is divided into 1,000 size-ordered bins around the circumference with each bin representing 0.1% of the 386,380,003 bp assembly. The distribution of scaffold lengths is shown in dark grey with the plot radius scaled to the longest scaffold present in the assembly (23,150,991 bp, shown in red). Orange and pale-orange arcs show the N50 and N90 scaffold lengths (13,374,257 and 9,210,517 bp), respectively. The pale grey spiral shows the cumulative scaffold count on a log scale with white scale lines showing successive orders of magnitude. The blue and pale-blue area around the outside of the plot shows the distribution of GC, AT and N percentages in the same bins as the inner plot. A summary of complete, fragmented, duplicated and missing BUSCO genes in the lepidoptera_odb10 set is shown in the top right. An interactive version of this figure is available at https://blobtoolkit.genomehubs.org/view/ilDiaChry1.1/dataset/CAKOAH01.1/snail.

The final assembly has a total length of 386.4 Mb in 41 sequence scaffolds with a scaffold N50 of 13.4 Mb ( Table 1). Most (99.89%) of the assembly sequence was assigned to 31 chromosomal-level scaffolds, representing 30 autosomes and the Z sex chromosome. Chromosome-scale scaffolds confirmed by the Hi-C data are named in order of size ( Figure 2– Figure 5; Table 2). The assembly has a BUSCO v5.3.2 ( Manni *et al.*, 2021) completeness of 99.1% (single 98.8%, duplicated 0.2%) using the lepidoptera_odb10 reference set. While not fully phased, the assembly deposited is of one haplotype. Contigs corresponding to the second haplotype have also been deposited.

**Table 1.** Genome data for *Diachrysia chrysitis*, ilDiaChry1.1.

Project accession data		
Assembly identifier	ilDiaChry1.1	
Species	*Diachrysia chrysitis*	
Specimen	ilDiaChry1	
NCBI taxonomy ID	179674	
BioProject	PRJEB50737	
BioSample ID	SAMEA8603181	
Isolate information	ilDiaChry1; male: thorax (PacBio), head (Hi-C)	
Assembly metrics *		*Benchmark*
Consensus quality (QV)	67.1	*≥ 50*
*k*-mer completeness	100%	*≥ 95%*
BUSCO **	C:99.1%[S:98.8%,D:0.2%], F:0.2%,M:0.8%,n:5,286	*C ≥ 95%*
Percentage of assembly mapped to chromosomes	99.89%	*≥ 95%*
Sex chromosomes	Z chromosome	*localised homologous pairs*
Organelles	Mitochondrial genome assembled	*complete single alleles*
Raw data accessions		
PacificBiosciences SEQUEL II	ERR8575372	
Hi-C Illumina	ERR8571654	
Genome assembly		
Assembly accession	GCA_932294365.1	
*Accession of alternate haplotype*	GCA_932294375.1	
Span (Mb)	386.4	
Number of contigs	42	
Contig N50 length (Mb)	13.3	
Number of scaffolds	41	
Scaffold N50 length (Mb)	13.4	
Longest scaffold (Mb)	23.2	
Genome annotation		
Number of protein-coding genes	18,320	
Number of gene transcripts	18,552	

* Assembly metric benchmarks are adapted from column VGP-2020 of “Table 1: Proposed standards and metrics for defining genome assembly quality” from ( Rhie *et al.*, 2021). ** BUSCO scores based on the lepidoptera_odb10 BUSCO set using v5.3.2. C = complete [S = single copy, D = duplicated], F = fragmented, M = missing, n = number of orthologues in comparison. A full set of BUSCO scores is available at https://blobtoolkit.genomehubs.org/view/ilDiaChry1.1/dataset/CAKOAH01.1/busco.

**Figure 3.**
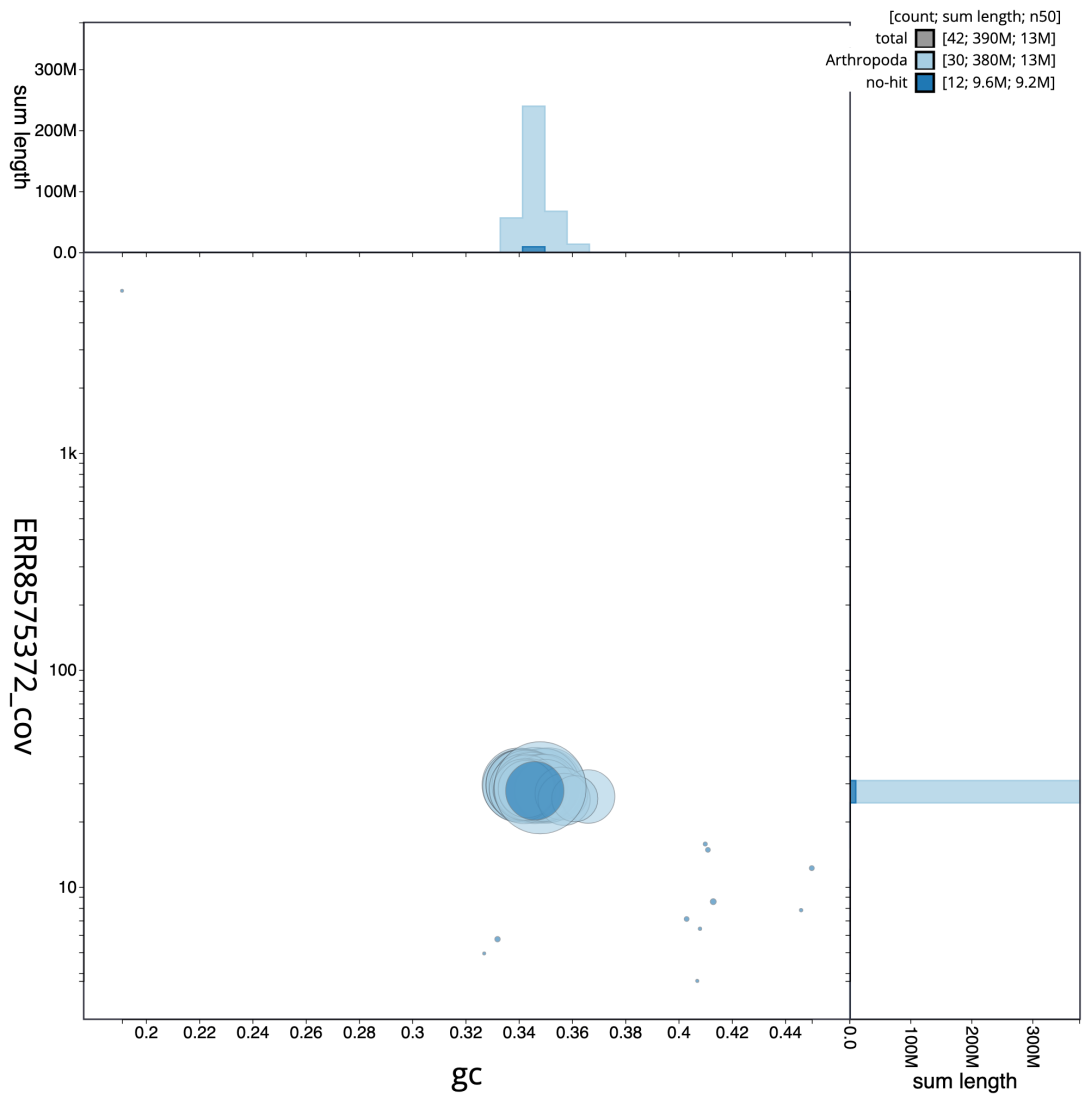
Genome assembly of *Diachrysia chrysitis*, ilDiaChry1.1: GC coverage. BlobToolKit GC-coverage plot. Scaffolds are coloured by phylum. Circles are sized in proportion to scaffold length. Histograms show the distribution of scaffold length sum along each axis. An interactive version of this figure is available at https://blobtoolkit.genomehubs.org/view/ilDiaChry1.1/dataset/CAKOAH01.1/blob.

**Figure 4.**
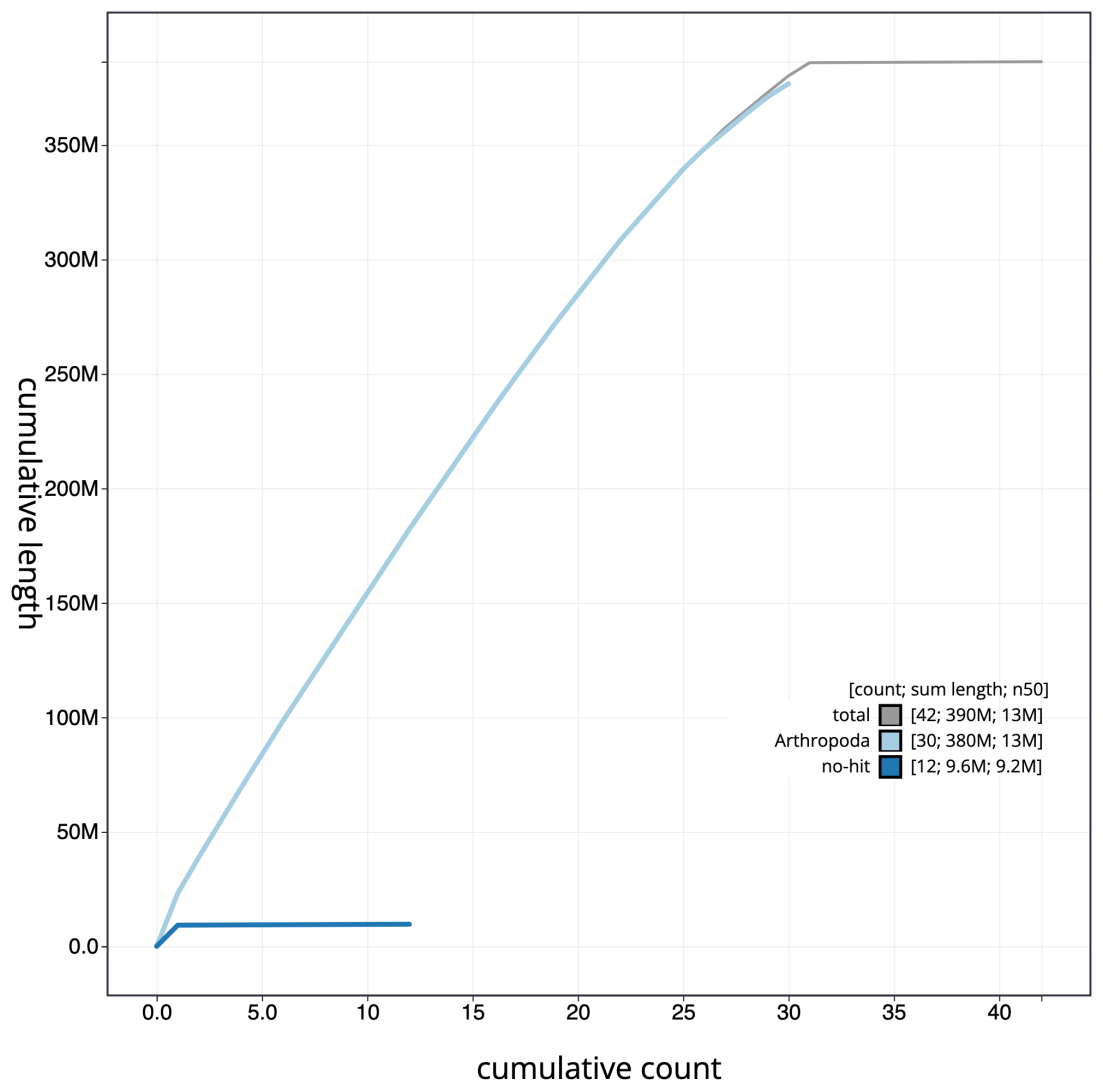
Genome assembly of *Diachrysia chrysitis*, ilDiaChry1.1: cumulative sequence. BlobToolKit cumulative sequence plot. The grey line shows cumulative length for all scaffolds. Coloured lines show cumulative lengths of scaffolds assigned to each phylum using the buscogenes taxrule. An interactive version of this figure is available at https://blobtoolkit.genomehubs.org/view/ilDiaChry1.1/dataset/CAKOAH01.1/cumulative.

**Figure 5.**
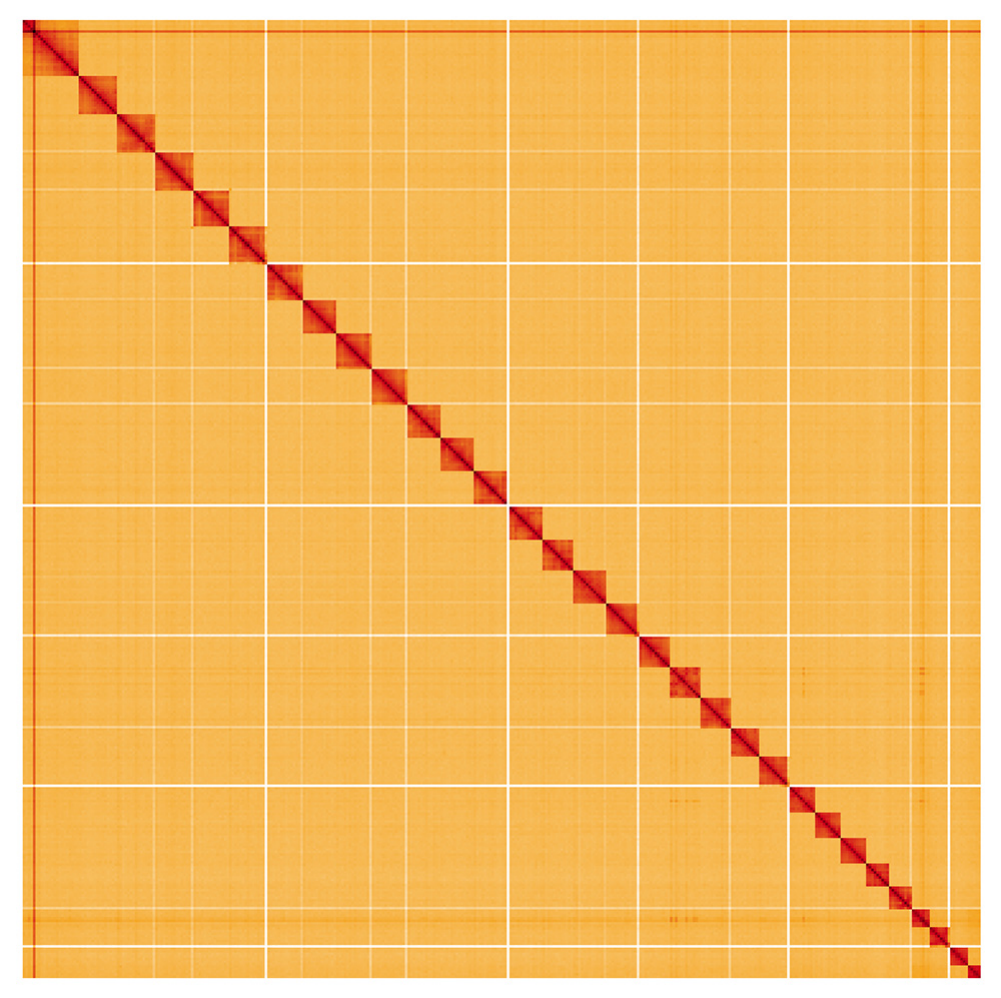
Genome assembly of *Diachrysia chrysitis*, ilDiaChry1.1: Hi-C contact map. Hi-C contact map of the ilDiaChry1.1 assembly, visualised using HiGlass. Chromosomes are shown in order of size from left to right and top to bottom. An interactive version of this figure may be viewed at https://genome-note-higlass.tol.sanger.ac.uk/l/?d=P3hH27lEToyfliwPcKQOBg.

**Table 2.** Chromosomal pseudomolecules in the genome assembly of *Diachrysia chrysitis*, ilDiaChry1.

INSDC accession	Chromosome	Size (Mb)	GC%
OW028642.1	1	15.75	34.6
OW028643.1	2	15.12	35
OW028644.1	3	15.12	35.1
OW028645.1	4	14.72	34
OW028646.1	5	14.68	34.9
OW028647.1	6	14.16	34
OW028648.1	7	14.01	34.2
OW028649.1	8	14	34.2
OW028650.1	9	13.97	34.4
OW028651.1	10	13.91	34.1
OW028652.1	11	13.64	34.1
OW028653.1	12	13.37	34.5
OW028654.1	13	13.32	34.4
OW028655.1	14	13.24	34.3
OW028656.1	15	13.19	34.2
OW028657.1	16	12.73	34.7
OW028658.1	17	12.53	34.5
OW028659.1	18	12.44	34.8
OW028660.1	19	11.96	34.9
OW028661.1	20	11.74	35
OW028662.1	21	11.52	34.3
OW028663.1	22	10.51	35
OW028664.1	23	10.44	34.4
OW028665.1	24	10.25	34.2
OW028666.1	25	9.21	34.6
OW028667.1	26	8.92	34.3
OW028668.1	27	7.75	36.6
OW028669.1	28	7.53	35.6
OW028670.1	29	7.34	35.7
OW028671.1	30	5.74	36.1
OW028641.1	Z	23.15	34.8
OW028672.1	MT	0.02	19.4

### Genome annotation report

The *D. chrysitis* GCA_932294365.1 genome assembly was annotated using the Ensembl rapid annotation pipeline ( Table 1; https://rapid.ensembl.org/Diachrysia_chrysitis_GCA_932294365.1/). The resulting annotation includes 18,552 transcribed mRNAs from 18,320 protein-coding genes.

## Methods

### Sample acquisition and nucleic acid extraction

One male *Diachrysia chrysitis* (ilDiaChry1) specimen was collected in Wytham Woods, Oxfordshire (biological vice-county: Berkshire), UK (latitude 51.77, longitude –1.34) on 8 September 2020, using a light trap. The specimen was collected and identified by Douglas Boyes (University of Oxford) and snap-frozen on dry ice.

DNA was extracted at the Tree of Life laboratory, Wellcome Sanger Institute (WSI). The ilDiaChry1 sample was weighed and dissected on dry ice with tissue set aside for Hi-C sequencing. Thorax tissue was cryogenically disrupted to a fine powder using a Covaris cryoPREP Automated Dry Pulveriser, receiving multiple impacts. High molecular weight (HMW) DNA was extracted using the Qiagen MagAttract HMW DNA extraction kit. HMW DNA was sheared into an average fragment size of 12–20 kb in a Megaruptor 3 system with speed setting 30. Sheared DNA was purified by solid-phase reversible immobilisation using AMPure PB beads with a 1.8X ratio of beads to sample to remove the shorter fragments and concentrate the DNA sample. The concentration of the sheared and purified DNA was assessed using a Nanodrop spectrophotometer and Qubit Fluorometer and Qubit dsDNA High Sensitivity Assay kit. Fragment size distribution was evaluated by running the sample on the FemtoPulse system.

### Sequencing

Pacific Biosciences HiFi circular consensus DNA sequencing libraries were constructed according to the manufacturers’ instructions. DNA sequencing was performed by the Scientific Operations core at the WSI on the Pacific Biosciences SEQUEL II (HiFi) instrument. Hi-C data were also generated from head tissue of ilDiaChry1 using the Arima v2 kit and sequenced on the Illumina NovaSeq 6000 instrument.

### Genome assembly

Assembly was carried out with Hifiasm ( Cheng *et al.*, 2021) and haplotypic duplication was identified and removed with purge_dups ( Guan *et al.*, 2020). The assembly was scaffolded with Hi-C data ( Rao *et al.*, 2014) using YaHS ( Zhou *et al.*, 2023). The assembly was checked for contamination as described previously ( Howe *et al.*, 2021). Manual curation was performed using HiGlass ( Kerpedjiev *et al.*, 2018) and Pretext ( Harry, 2022). The mitochondrial genome was assembled using MitoHiFi ( Uliano-Silva *et al.*, 2022), which performed annotation using MitoFinder ( Allio *et al.*, 2020). The genome was analysed and BUSCO scores were generated within the BlobToolKit environment ( Challis *et al.*, 2020). Table 3 contains a list of all software tool versions used, where appropriate.

**Table 3.** Software tools and versions used.

Software tool	Version	Source
BlobToolKit	3.5.0	Challis *et al.*, 2020
Hifiasm	0.16.1-r375	Cheng *et al.*, 2021
HiGlass	1.11.6	Kerpedjiev *et al.*, 2018
MitoHiFi	1	Uliano-Silva *et al.*, 2022
PretextView	0.2	Harry, 2022
purge_dups	1.2.3	Guan *et al.*, 2020
YaHS	yahs-1.1.91eebc2	Zhou *et al.*, 2023

### Genome annotation

The BRAKER2 pipeline ( Brůna *et al.*, 2021) was used in the default protein mode to generate annotation for the *Diachrysia chrysitis* assembly (GCA_934047225.1) in Ensembl Rapid Release.

### Ethics and compliance issues

The materials that have contributed to this genome note have been supplied by a Darwin Tree of Life Partner. The submission of materials by a Darwin Tree of Life Partner is subject to the Darwin Tree of Life Project Sampling Code of Practice. By agreeing with and signing up to the Sampling Code of Practice, the Darwin Tree of Life Partner agrees they will meet the legal and ethical requirements and standards set out within this document in respect of all samples acquired for, and supplied to, the Darwin Tree of Life Project. All efforts are undertaken to minimise the suffering of animals used for sequencing. Each transfer of samples is further undertaken according to a Research Collaboration Agreement or Material Transfer Agreement entered into by the Darwin Tree of Life Partner, Genome Research Limited (operating as the Wellcome Sanger Institute), and in some circumstances other Darwin Tree of Life collaborators.

## Data Availability

European Nucleotide Archive: *Diachrysia chrysitis* (burnished brass). Accession number PRJEB50737; https://identifiers.org/ena.embl/PRJEB50737. ( Wellcome Sanger Institute, 2022) The genome sequence is released openly for reuse. The *Diachrysia chrysitis* genome sequencing initiative is part of the Darwin Tree of Life (DToL) project. All raw sequence data and the assembly have been deposited in INSDC databases. Raw data and assembly accession identifiers are reported in Table 1.

## Author information

Members of the University of Oxford and Wytham Woods Genome Acquisition Lab are listed here: https://doi.org/10.5281/zenodo.4789928.

Members of the Darwin Tree of Life Barcoding collective are listed here: https://doi.org/10.5281/zenodo.4893703.

Members of the Wellcome Sanger Institute Tree of Life programme are listed here: https://doi.org/10.5281/zenodo.4783585.

Members of Wellcome Sanger Institute Scientific Operations: DNA Pipelines collective are listed here: https://doi.org/10.5281/zenodo.4790455.

Members of the Tree of Life Core Informatics collective are listed here: https://doi.org/10.5281/zenodo.5013541.

Members of the Darwin Tree of Life Consortium are listed here: https://doi.org/10.5281/zenodo.4783558.
