# Supplementary material for: The genome sequence of the Burnished Brass, Diachrysia chrysitis (Linnaeus, 1758)
Source: Wellcome Open Res. 2023 Feb 15;8:82. [Version 1] doi: 10.12688/wellcomeopenres.18990.1 (PMC10357075; doi:10.12688/wellcomeopenres.18990.1)
Supplement: Peer review history

# Peer review: Reviewer response for version 1

**DOI:** 10.21956/wellcomeopenres.21054.r61851

**Published:** 2023-07-19

**Authors:** Bernard Moussian

**Affiliations:** 1 Universite Cote d'Azur, Nice, Provence-Alpes-Côte d'Azur, France

Douglas Boyes has a long-standing expertise on lepidopteran genome sequencing and evaluation. It was a pleasure reading the manuscript. The data is of high quality.

 In the short text, there are only two sentences that needs attention. On page 4, it is stated that “The resulting annotation includes 18,552 transcribed mRNAs from 18,320 protein-coding genes.”. First, this is a low number (in *Drosophila* more than 45% of genes are alternatively spliced); second, no transcription was anaylsed, therefore, the wording should be different in my opinion, as there may be pseudo-genes etc. On page 5, the author names himself as the one who identified the species. How was it done? Based on which criteria?

Are sufficient details of methods and materials provided to allow replication by others?

Yes

Is the rationale for creating the dataset(s) clearly described?

Yes

Are the datasets clearly presented in a useable and accessible format?

Partly

Are the protocols appropriate and is the work technically sound?

Yes

Reviewer Expertise:

Insect genetics and molecular ecology

I confirm that I have read this submission and believe that I have an appropriate level of expertise to confirm that it is of an acceptable scientific standard.

# Peer review: Reviewer response for version 1

**DOI:** 10.21956/wellcomeopenres.21054.r61860

**Published:** 2023-07-19

**Authors:** Yue Ma

**Affiliations:** 1 Zhejiang Normal University, Jinhua, China

The paper presents the genome assembly from an individual male **of *Diachrysia chrysitis *(the Burnished Brass; Arthropoda; Insecta; Lepidoptera; Noctuidae) plus with its mitochondrial genome. The authors collected the specimen using light trap, the results are solid, the methods are also rational, providing additional valuable molecular data for Noctuidae moths. Thus, I recommend its indexing.

Are sufficient details of methods and materials provided to allow replication by others?

Yes

Is the rationale for creating the dataset(s) clearly described?

Yes

Are the datasets clearly presented in a useable and accessible format?

Yes

Are the protocols appropriate and is the work technically sound?

Yes

Reviewer Expertise:

entomology; phylogenetics; macroevolutionary dynamics

I confirm that I have read this submission and believe that I have an appropriate level of expertise to confirm that it is of an acceptable scientific standard.
